# Prospective Randomized Controlled Trial on the Effects of Almonds on Facial Wrinkles and Pigmentation

**DOI:** 10.3390/nu13030785

**Published:** 2021-02-27

**Authors:** Iryna Rybak, Alexis E. Carrington, Simran Dhaliwal, Aliza Hasan, Hera Wu, Waqas Burney, Jessica Maloh, Raja K. Sivamani

**Affiliations:** 1 Department of Dermatology, University of California-Davis, Sacramento, CA 95816, USA; irybak@ucdavis.edu (I.R.); aecarrington@ucdavis.edu (A.E.C.); simkdhal@gmail.com (S.D.); malohjessica@gmail.com (J.M.); 2 Department of Dermatology, Southern Illinois University, Springfield, IL 62901, USA; ahasan57@siumed.edu; 3 College of Medicine, California Northstate University, Sacramento, CA 95757, USA; herawu@gmail.com; 4 Department of Biological Sciences, California State University, Sacramento, CA 90802, USA; waqas.ahmed.burney@gmail.com; 5 Pacific Skin Institute, Sacramento, CA 95815, USA; 6 Zen Dermatology, Sacramento, CA 95819, USA

**Keywords:** almonds, aging, skin aging, vitamin E, tocopherol, wrinkles

## Abstract

Background: Almonds have long been studied as a rich source of fatty acids, phytochemical polyphenols and antioxidants such as vitamin E. A recent study compared almond supplementations to a calorie-matched intervention for 16 weeks, yielding statistically significant improvement in wrinkle severity in postmenopausal women with Fitzpatrick skin types I and II that received almonds. This study furthers that assessment with a larger population and duration of 24 weeks to assess the influence of almond consumption on wrinkle severity, skin pigmentation and other skin biophysical profiles. Objective: To investigate the effects of almond consumption on photoaging such as wrinkles and pigment intensity as well as facial biophysical parameters such as sebum production, skin hydration and water loss. Design and interventions: A prospective, randomized controlled study assessed postmenopausal women with Fitzpatrick skin types I or II who consumed 20% of their daily energy consumption in either almonds or a calorie-matched snack for 24 weeks. A facial photograph and image analysis system was used to obtain standardized high-resolution photographs and information on wrinkle width and severity at 0, 8, 16 and 24 weeks. Measurements of transepidermal water loss (TEWL), skin pigmentation, skin hydration and sebum production were also completed at each visit. Results: The average wrinkle severity was significantly decreased in the almond intervention group at week 16 and week 24 compared to baseline by 15% and 16%, respectively. Facial pigment intensity was decreased 20% in the almond group at week 16 and this was maintained by week 24. There were no significant differences in skin hydration or TEWL in the almond group compared to the control, although sebum excretion was increased in the control group. Conclusion: The daily consumption of almonds may improve several aspects of photoaging such as facial wrinkles and pigment intensity in postmenopausal women. In conclusion, the daily consumption of almonds may contribute to the improvement of facial wrinkles and reduction of skin pigmentation among postmenopausal women with Fitzpatrick skin types I and II.

## 1. Introduction

Nutraceuticals and food-based cosmetics are a growing field in dermatology. Almonds are a commonly studied food, as they are a rich source of fatty acids, phytochemical polyphenols and antioxidants such as vitamin E [1]. A previous randomized 16-week pilot study that compared almond ingestion to a calorie-matched intervention showed, with statistical significance, that almond supplementation improved wrinkle severity in postmenopausal women with Fitzpatrick skin types I and II, relative to control [2].

Postmenopausal women are more susceptible to the development of facial-aging related changes [3,4]. Skin aging is a multifactorial process and there is growing evidence that nutrition levels and eating habits can contribute to morphological changes in the skin and modulate oxidative stress and inflammation [5]. In the case of almonds, the vitamin E (alpha-tocopherol) content can range between 21.9–31mg/100g of almonds [6] and it has been shown that almond consumption can elevate circulating alpha-tocopherols [7,8]. Additionally, previous studies have shown that the ingestion of alpha-tocopherol-containing supplements can improve facial wrinkles in post-menopausal women [9,10]. Alpha-tocopherol has antioxidant and photoprotective functions, especially against ultraviolet type A radiation that is implicated in the development of wrinkles and pigment unevenness [11]. Furthermore, tocopherol has pigment reducing and skin brightening properties [12,13].

The Fitzpatrick scale is a classification system that ranges from I–VI and was developed to estimate the response to ultraviolet exposure with varying skin pigmentation [14]. The type I classification refers to skin that always burns and never tans, while the type II classification refers to skin that burns frequently and tans with difficulty [14]. In addition to estimating the response to ultraviolet light, some research suggests that in middle-aged subjects, Fitzpatrick skin types may be influential in skin aging, where skin types I and II may experience photoaging at an accelerated pace [15,16]. Fitzpatrick types I and II were the population of interest in the pilot study, and the population assessed in this investigation.

This study expanded upon the previous pilot study to both expand the number of participants and to increase the assessment follow up from 16 weeks to 24 weeks. We assessed the influence of almond consumption on wrinkle severity, skin pigmentation and other skin biophysical properties.

## 2. Materials and Methods

### 2.1. Study Participants

This study was conducted from September 2018 to February 2020 as a randomized, investigator blinded, 24-week study. The study was approved by the Institutional Review Board at the University of California, Davis and registered on ClinicalTrials.gov (NCT03729700). All participants provided written informed consent prior to participation in the study. 91 healthy females were recruited and screened for eligibility at the UC Davis Dermatology clinic. Participants were included if they were postmenopausal women with a Fitzpatrick skin type I or II. Participants were excluded if they had a nut allergy, an autoimmune photosensitive condition, or a known genetic condition with a deficiency in collagen production. Participants were also excluded if they already obtained at least 20% of their energy intake from nut consumption and those with implausible reported energy intakes of <1000 kcal/d or >3000 kcal/d throughout the study (Table 1). Individuals were required to discontinue high antioxidant supplements and daily food sources during a washout period prior to participation, which included: antioxidant supplements, beverages with added vitamins, nutrition bars with high added vitamin E, omega-3 fatty acid supplements, herbal supplements, sunflower seeds, nuts, nut butter, nut milks, or nut oil intake other than that provided by the studies. Current smokers, those that have smoked within the past year, and former smokers with greater than a 20 pack-year history of smoking within the past 20 years were also excluded [1]. All participants were restricted in the use of topical ingredients such as retinoids (tretinoin, adapalene, or retinol), vitamin C, vitamin E, or any nut-based oils or extracts during the study and in the two weeks prior to study participation.

### 2.2. Study Design and Intervention

**Figure 1:**
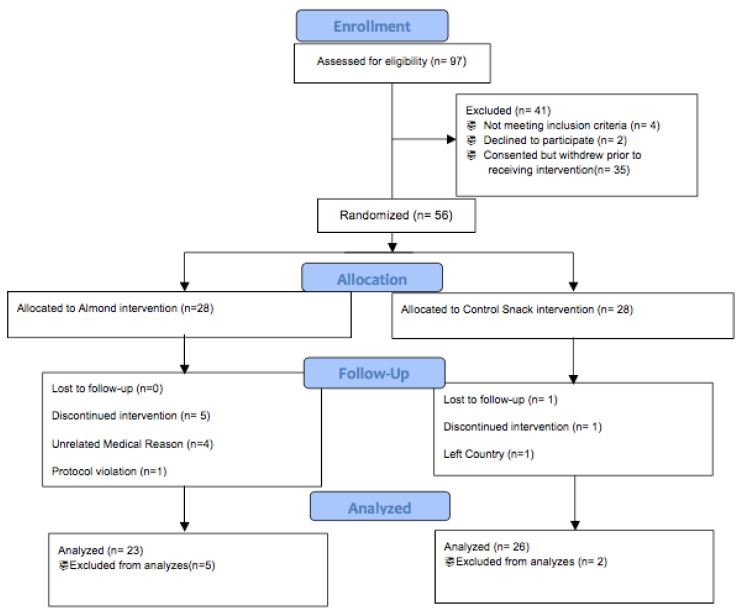
CONSORT (Consolidated Standards of Reporting Trials) flow diagram.

Each subject participated over a period of 24 weeks, which consisted of a total of eight study visits. 91 eligible participants were screened and a total of 56 females were randomized into two intervention groups: almond group (ages: 51–77; 28 females) and control group (ages: 47–84; 28 females); (see Figure 1 for CONSORT (Consolidated Standards of Reporting Trials) diagram). All supplement assignment groups were randomized and pre-allocated using a computer-based randomization generator with blinded sealed envelopes. The participants were enrolled and assigned into one of the groups by the clinical research coordinator. The participants were weighed at each visit (Table 2). The two intervention groups consisted of those receiving almonds and those who received a calorie-matched snack. The almond dose was provided as 20% of the total energy in the diet and this dose was chosen based on previous almond-based pilot clinical study data. The almond snack group received approximately 2 g of sugars per day whereas the control snack group received approximately 8 g of sugars per day that was additional to their regular food intake. The control snack was commercially available, individually wrapped food products including pretzels, granola bars, and fig bars (Table 3).

### 2.3. Facial Imaging and Skin Biophysical Measures

**Figure 2:**
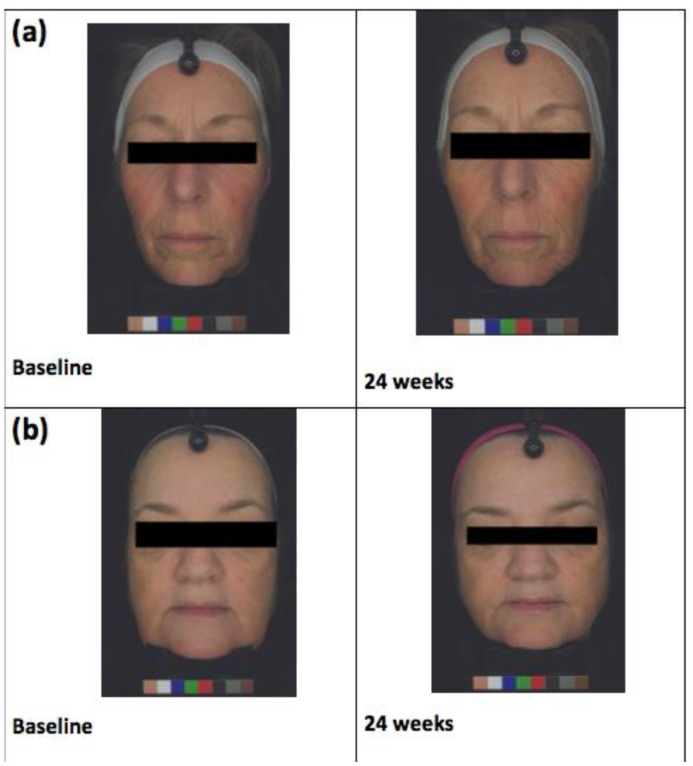
(**a**) Almond subject and (**b**) control subject facial images at baseline and at 24 weeks of receiving the almond or snack interventions, respectively.

The BTBP 3D Clarity Pro^®^ Facial Modeling and Analysis System (Brigh-Tex BioPhotonics, San Jose, CA, USA) was utilized to obtain high-resolution facial photographs for all study participants at baseline, 8, 16, and 24 weeks (Figure 2). Facial wrinkles were then analyzed through a computer algorithm-based model that incorporates the measurement of the wrinkles’ depth and width. Image analysis was also used to assess the average pigment intensity across the entire face. The following skin biophysical measures were assessed: sebum production (Sebumeter^®^ SM 815; Courage and Khazaka, Cologne, Germany), transepidermal water loss (TEWL) (Vapometer; Delfin Technologies, Stamford, CT, USA), and skin hydration (MoistureMeterSC; Delfin Technologies, Stamford, CT, USA) at baseline, 8, 16, and 24 weeks. Subjects were asked to report any adverse effects throughout the study.

### 2.4. Statistical Analysis

A priori power analysis based on the previous pilot study results showed that there was greater than 90% power to detect a 10% difference in wrinkle severity between the almond and control groups at Week 16, with the recruitment of at least 20 subjects in each group with alpha set to 0.05. All data were assessed with an intention to treat analysis by including all subjects that were enrolled in the trial and had received any study intervention. Statistical analyses were performed using paired *t* tests (or Wilcoxon signed-rank test for nonparametric measures) with correction for repeated measures. *p*-values less than 0.05 and false discovery *Q* values less than 0.2 were considered significant.

## 3. Results

Out of 91 participants who were screened, 56 postmenopausal females met the enrollment criteria and were randomized into an almond intervention group (*n* = 28) or a controlled, calorie-matched snack group (*n* = 28). Of the 28 that were randomized, 23 of the women in the almond supplementation group and 26 of the women in the control supplementation group completed the study.

### 3.1. Computer-Based Photographic Analysis of Facial Features

**Figure 3:**
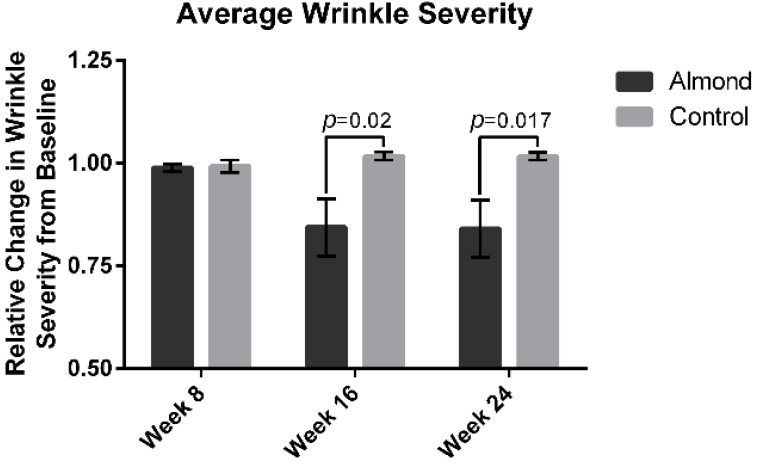
Computer-based photographic analysis of wrinkle severity was significantly decreased in the almond intervention group compared with the control intervention by 15% and 16% at week 16 and 24, respectively. Significant p values are noted on the figure. Error bars represent the standard error of the mean (SEM).

**Figure 4:**
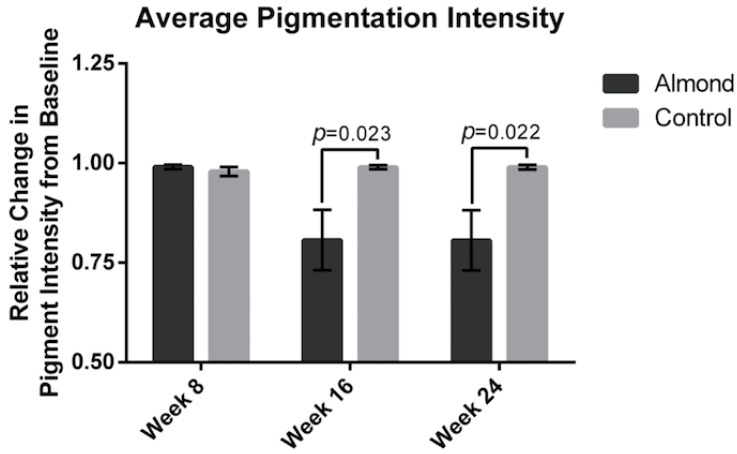
Relative change in skin pigment intensity was measured showing a 20% reduction from baseline in the almond group at both week 16 and 24. Significant p values are noted on the figure. Error bars represent SEM.

The average wrinkle severity (Figure 3) was significantly decreased in the almond group by 15% at week 16 and 16% at week 24 compared to the baseline (*p* < 0.05). There was no improvement in the wrinkle severity in the control supplementation group. The average facial pigment intensity was decreased by 20% at week 16 in the almond supplementation group (Figure 4) and remained at 20% at week 24. There was no improvement in facial pigment intensity in the control supplementation group.

### 3.2. Skin Hydration

**Figure 5:**
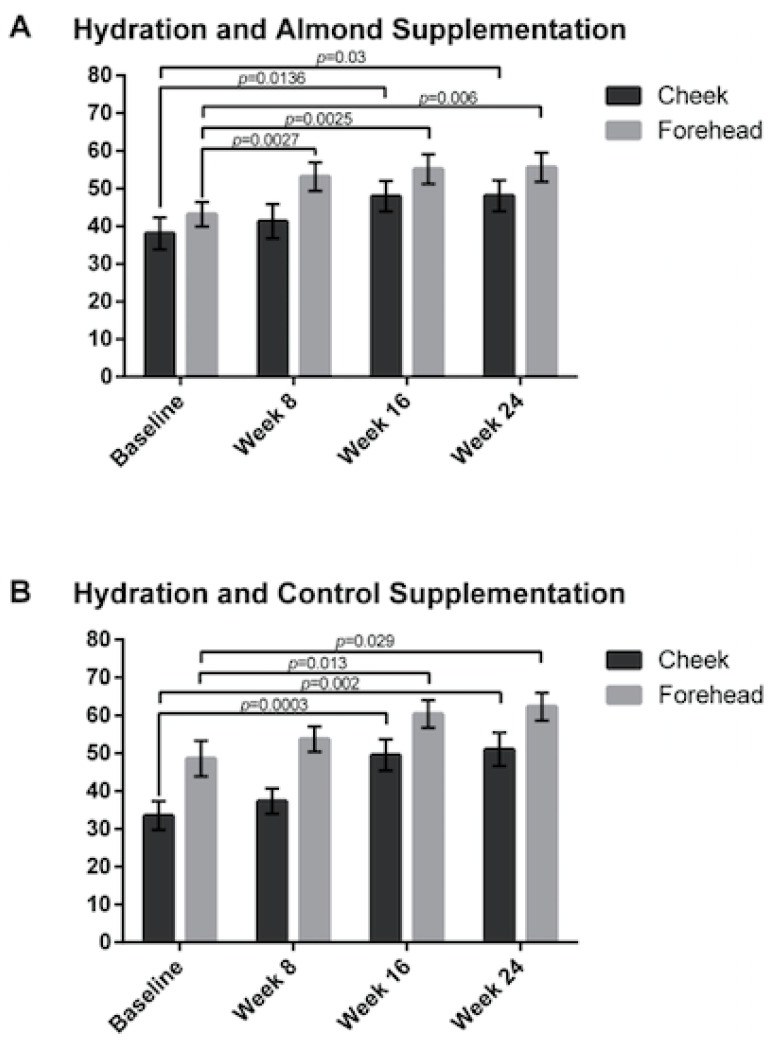
Skin hydration was measured resulting in no significant difference between the (**A**) almond and (**B**) control groups. Significant p values are noted on the figure. Error bars represent SEM.

Skin hydration was increased in both the control and almond groups from baseline on both the cheek and the forehead (Figure 5).

### 3.3. Transepidermal Water Loss (TEWL)

**Figure 6:**
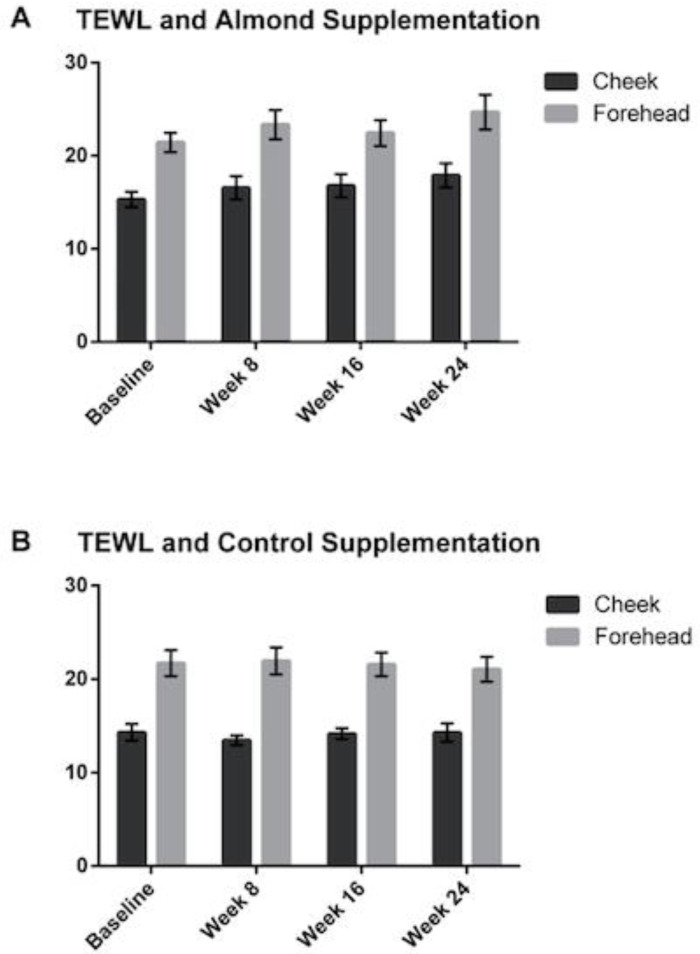
Changes in transepidermal water loss were measured at baseline, 8, 16 and 24 weeks, showing no significant differences detected between the (**A**) almond and (**B**) control groups. Error bars represent SEM.

There were no significant changes in TEWL on the foreheads or cheeks in either the almond or control groups at any time point compared to baseline (Figure 6).

### 3.4. Sebum Excretion

**Figure 7:**
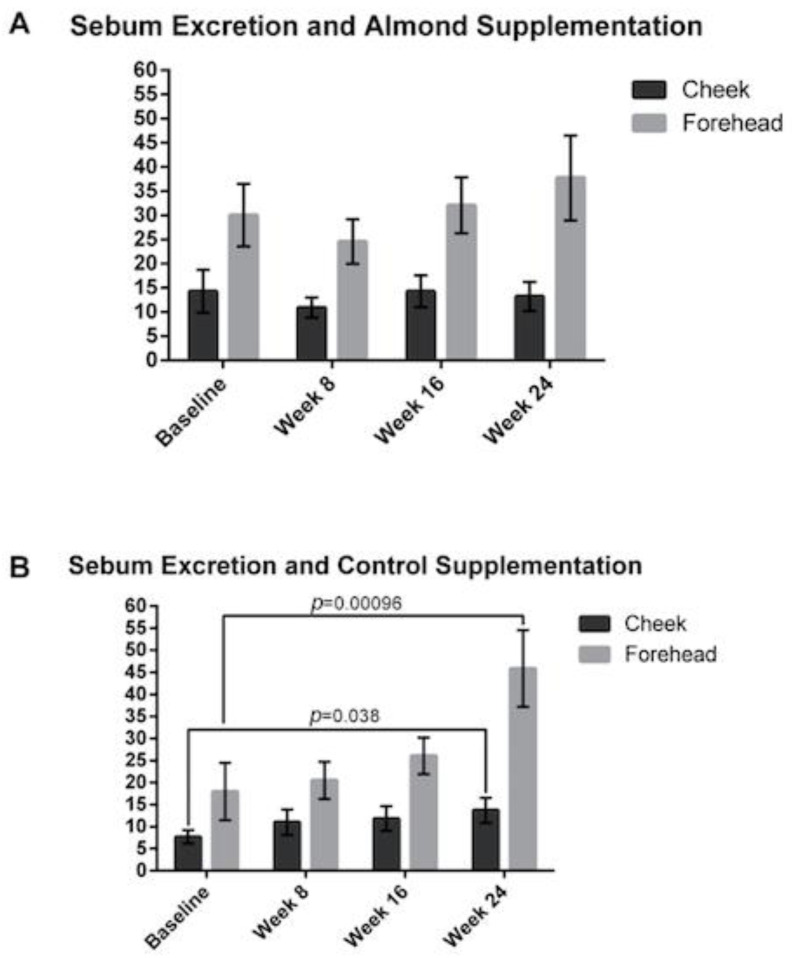
Changes in sebum excretion of the forehead and cheek were measured at baseline, 8, 16 and 24 weeks with (**A**) almond and (**B**) control supplementation. Sebum excretion rates were increased in the control supplementation group but not in the almond supplementation group. Significant p values are noted on the figure. Error bars represent SEM.

The sebum excretion on the forehead was significantly higher compared to the cheeks in both the almond and control groups. Interestingly, there was no significant change in sebum excretion in the almond group (Figure 7). However, there was a significant increase in the forehead sebum excretion rate for participants in the control supplementation group with a 45% and 155% increase at weeks 16 and 24, respectively (*p* < 0.05).

## 4. Discussion

This randomized clinical trial demonstrates a significant reduction in wrinkle severity in the intervention group consuming almonds. In fact, this study showed an increased magnitude of reduction in wrinkle severity of 15% at 16 weeks, compared to the previous pilot study assessing the same outcome, which showed 9% reduction at 16 weeks compared to baseline [2]. Furthermore, this study showed a continued reduction of that magnitude at 24 weeks. Surprisingly, there was also a reduction in the overall pigment intensity in the almond supplementation group.

The antioxidant and photoprotective properties from alpha-tocopherols in almonds may serve to reduce development of wrinkles and pigment unevenness [11]. This may further account for the results in the reduction of skin pigmentation in the almond group. Vitamin E’s effects on pigmentation has been documented in the literature, especially by interacting with lipid peroxidation of melanocyte membranes, increasing glutathione content and inhibiting tyrosinase, a key enzyme in pigmentation [17]. Interestingly, vitamin E was found to have minimal efficacy in treating pigmented disease such as chloasma [18]; however, when used in combination with other vitamins, such as A and C, vitamin E showed an improvement in skin pigmentation [18,19]. Interestingly, almonds are not just a rich source of vitamin E but also a source of niacin, which has been shown to improve facial pigmentation. Our findings emphasize the need to look at almonds as a whole food with multiple nutrient components rather than oversimplifying its nutritional content to its tocopherol content.

This study confirmed the findings of a previous almond pilot study in regards to the TEWL and sebum excretion results [2], except that the control group had a large increase in their sebum excretion rate. Our findings suggest that the caloric intake may not be the driving factor in sebum excretion rate. The food supplementation in the control group added extra sugars to the diet compared to the almond supplementation group and may explain why the sebum excretion rate was increased in the control supplementation group, although this would require further specific study. Although previous research has demonstrated that sebum excretion has a negative association with wrinkle severity [20], our data suggests that sebum excretion rate may be more nuanced based on the foods that are ingested and that the role of the sugar content in the foods may influence wrinkles, as was suggested in a previous prospective study with high mango intake compared to low mango intake [21].

The influence of whole foods on skin health and appearance continues to be a growing field. Recently, a study assessed the influence of mangos on wrinkles and skin pigmentation [21]. Specifically, Fam et. al. found an increase in erythema of the cheeks with 85 g of mango intake (*p* = 0.04), reduction of wrinkles with 85 g of mangos and the opposite effects with a 250 g mango intake in postmenopausal women with Fitzpatrick skin types II or III. It is thought that ß-carotene and other carotenoids provide photoprotection of the skin through antioxidant effects [21]. Therefore, it is unlikely that erythema was caused by the amount of β-carotene consumed from the fruit. Furthermore, β-carotene has been shown to decrease erythema in other studies [22,23].

Consistent results of the reduction of wrinkles due to almonds in this and the previous pilot study suggest that diet can influence markers of facial photoaging. In addition to basic sun protection habits, such as regular use of mineral-based sunscreens and sun protective clothing, almonds and their components may have a part in the reduction of skin wrinkles and pigment intensity. Our unexpected findings of reduced pigment intensity suggest that future studies should include higher Fitzpatrick skin types and younger participants to further assess the role of almond supplementation on facial pigment.

### Limitations

This study was limited to 24 weeks and our results do not give insight into longer durations of ingestion. This study was limited to postmenopausal woman with sun sensitive Fitzpatrick skin types I and II and the results cannot be generalized to younger, male, or higher Fitzpatrick skin type populations. In addition, the supplementation between the almond vs. the control group was not macronutrient matched, though it was calorically matched.

## 5. Conclusions

In conclusion, the daily consumption of almonds may be an effective contributor to improving facial wrinkles and reducing skin pigmentation among postmenopausal women with Fitzpatrick skin types I and II. Further studies should expand the study populations to participants that are younger and that have higher Fitzpatrick skin types.

## Figures and Tables

**Table 1:** Mean and standard deviation (SD) of age and caloric intake (in kilocalories) of participants in the control and almond group at each visit.

**CONTROL SNACK GROUP**					
	AGE	Baseline kcals	Week 8 kcals	Week 16 kcals	Week 24 kcals
Mean	65.14	1614.55	1506.87	1448.58	1527.28
SD	8.14	332.62	374.89	340.39	397.39
**ALMOND GROUP**					
	AGE	Baseline kcals	Week 8 kcals	Week 16 kcals	Week 24 kcals
Mean	61.72	1826.25	1811.45	1968.33	1893.35
SD	8.76	567.78	384.75	512.78	440.16

**Table 2:** Mean and standard deviation (SD) of the weight of participants in the control group and almond group (in pounds) at each visit.

**Control Group**		
Visit	Mean Weight (lbs)	SD
Baseline	158.06	45.34
Visit 8	159.86	45.90
Visit 16	159.54	44.11
Visit 24	159.65	44.97
**Almond Group**		
Visit	Mean Weight (lbs)	SD
Baseline	146.97	17.18
Visit 8	146.91	18.78
Visit 16	147.58	19.59
Visit 24	147.09	20.19

**Table 3:** Daily sugars, fat, and protein content of control and almond group supplementation.

	Control Group	Almond Group
Sugars per daily serving (g)	8	2
Fat per daily serving (g)	4	28
Protein per daily serving (g)	7	20.3

## Data Availability

No publicly archived datasets.
